# Acidotolerant soil nitrite oxidizer “*Candidatus* Nitrobacter laanbroekii” NHB1 alleviates constraints on growth of acidophilic soil ammonia oxidizers

**DOI:** 10.1093/ismeco/ycaf244

**Published:** 2025-12-23

**Authors:** Eleftheria Bachtsevani, Linda Hink, Yiyu Meng, Christopher J Sedlacek, Sungeun Lee, Holger Daims, Michael Wagner, Cécile Gubry-Rangin, Wietse de Boer, Christina Hazard, James I Prosser, Graeme W Nicol

**Affiliations:** Université Claude Bernard Lyon 1, CNRS, INRAE, VetAgro Sup, Laboratoire d’Ecologie Microbienne, Villeurbanne 69622, France; Université Claude Bernard Lyon 1, CNRS, INRAE, VetAgro Sup, Laboratoire d’Ecologie Microbienne, Villeurbanne 69622, France; Institute of Microbiology, Leibniz University Hannover, 30419 Hannover, Germany; School of Biological Sciences, University of Aberdeen, Cruickshank Building, St Machar Drive, Aberdeen, AB24 3UU, Scotland, United Kingdom; Centre for Microbiology and Environmental Systems Science, University of Vienna, 1030 Vienna, Austria; Biology Department, University of Southern Indiana, Evansville, IN 47712, United States; Université Claude Bernard Lyon 1, CNRS, INRAE, VetAgro Sup, Laboratoire d’Ecologie Microbienne, Villeurbanne 69622, France; Centre for Microbiology and Environmental Systems Science, University of Vienna, 1030 Vienna, Austria; Centre for Microbiology and Environmental Systems Science, University of Vienna, 1030 Vienna, Austria; School of Biological Sciences, University of Aberdeen, Cruickshank Building, St Machar Drive, Aberdeen, AB24 3UU, Scotland, United Kingdom; Institute of Microbiology, Leibniz University Hannover, 30419 Hannover, Germany; Biology Department, University of Southern Indiana, Evansville, IN 47712, United States; Université Claude Bernard Lyon 1, CNRS, INRAE, VetAgro Sup, Laboratoire d’Ecologie Microbienne, Villeurbanne 69622, France; School of Biological Sciences, University of Aberdeen, Cruickshank Building, St Machar Drive, Aberdeen, AB24 3UU, Scotland, United Kingdom; Université Claude Bernard Lyon 1, CNRS, INRAE, VetAgro Sup, Laboratoire d’Ecologie Microbienne, Villeurbanne 69622, France

**Keywords:** Nitrobacter, nitrite-oxidizing bacteria, ammonia-oxidizing archaea, acidophilic, acidic soil, ammonia oxidation, nitrite oxidation, nitrification, cyanase

## Abstract

*Nitrobacter* strain NHB1 is a nitrite-oxidizing bacterium previously demonstrated to form a consortium capable of nitrification under acidic conditions when cocultivated with a neutrophilic ammonia-oxidizing bacterium. Here, we characterize the growth of isolated NHB1 under different pH and nitrite (NO_2_^−^) concentrations, as well as its influence on the activity of obligately acidophilic soil ammonia-oxidizing archaea (AOA) isolated from acidic soils when grown in coculture. NHB1 is acidotolerant with optimal growth at pH 6.0 (range: 5.0–7.5) at an initial NO_2_^−^ concentration of 500 μM. However, at lower NO_2_^−^ concentrations, closer to those found in soil, its pH optimum decreases to 5.0 and with detectable growth extended to pH 3.5. In coculture, NHB1 enhances the growth of the acidophilic AOA *Nitrosotalea devaniterrae* Nd1 and *Nitrosotalea sinensis* Nd2, which are highly sensitive to NO_2_^-^derived compounds and typically oxidize only ~200 to 300 μM ammonia (NH_3_) when grown in batch cultures as isolates. However, in coculture with NHB1, both strains oxidized up to ~3 mM NH_3_, limited only by the buffering capacity of the medium, and their pH range was also extended downward by ~0.5 units. NHB1 also possesses a cyanase, enabling reciprocal cross-feeding through cyanate-derived NH_3_ production while utilizing AOA-derived NO_2_^−^. These findings suggest that NO_2_^−^ removal is essential for ammonia oxidizer growth in acidic soils and emphasize the importance of considering substrate and metabolic product concentrations when characterizing ecophysiology. Genome analysis reveals that NHB1 is distinct from validated species, and we propose the name *“Nitrobacter laanbroekii.”*

## Introduction

Microbially mediated nitrification in soil is often driven by chemolithoautotrophs. Canonical ammonia-oxidizing archaea (AOA) of the class *Nitrososphaeria* [1] and ammonia-oxidizing bacteria (AOB) of the genera *Nitrosomonas*, *Nitrosospira*, and *Nitrosococcus* [2] catalyse the first step of nitrification, oxidizing ammonia (NH₃) to nitrite (NO_2_^−^) [3]. This process is coupled with the activity of nitrite-oxidizing bacteria (NOB), such as *Nitrobacter* and *Nitrospira*, which subsequently oxidize NO_2_^−^ to nitrate (NO_3_^−^) [4]. Additionally, recently discovered comammox *Nitrospira* are capable of oxidizing NH₃ directly to NO_3_^−^ and are ubiquitous in soil [5, 6].

While nitrification is typically studied in near-neutral soils, it also occurs across a broad pH range. In acidic soils, which represent ~30% of the world’s soils, ammonia oxidation was historically considered paradoxical due to the limited availability of NH₃ (pKa for NH₃/NH₄^+^ = 9.25) and the toxicity of nitrous acid (HNO₂) and its decomposition products at low pH [7, 8]. To sustain ammonia oxidizer growth in culture, buffers or neutralizing media have typically been required, and growth was initially thought to be possible only at pH ≥6.5. The subsequent discovery and cultivation of acidophilic and acidotolerant ammonia oxidizers primarily explained this paradox [9–12]. However, additional mechanisms—including ureolytic activity [13, 14], biofilm formation [15], and aggregation [16]—have been shown to support the survival of neutrophilic nitrifiers under acidic conditions [17]. Furthermore, since canonical ammonia oxidizers often coexist with NOB in soil, enabling mutualistic interactions, the cooperative activity of NOB may be crucial for ammonia oxidizer persistence in acidic environments.

AOA from the *Nitrosotalea* lineage are globally distributed and abundant in acidic soils [18]. The first isolates of this genus were *Nitrosotalea devaniterrae* Nd1, cultivated from a pH 4.5 agricultural soil [8], and *Nitrosotalea sinensis* Nd2, isolated from an acid sulphate paddy soil (pH 4.7) [19]. Both isolates are obligately acidophilic and grow within a pH range of 4.0–6.0. Despite being phylogenetically close, the two strains possess distinct physiological traits, such as contrasting specific growth rates and optimal growth temperatures. However, both isolates are inhibited by relatively low concentrations of NO_2_^−^ compared to other ammonia oxidizers with isolated culture growth being fully inhibited once a nitrite concentration of 200–300 μM is reached [9].

In addition to acidophilic AOA, the role of acidophilic/acid-tolerant NOB in supporting nitrification under low pH conditions is being increasingly recognized. *Nitrobacter* populations are a major component of soil NOB communities. While most isolates are grown at neutral pH [20], acidophilic/acidotolerant strains have been reported [16, 21–24]. These include *Nitrobacter* NHB1, which was originally isolated from pH 3.8 fertilized heathland soil together with the AOB *Nitrosospira* AHB1 [13]. Although neutrophilic when grown in isolation, AHB1 was active down to pH 4 when grown with NHB1 [25] and neutrophilic *Nitrosospira*-like bacteria enriched from acidic forest soil grew at pH 4 when surrounded by *Nitrobacter*-like bacteria in aggregates [16]. Acidotolerant NOB may therefore have a role in protecting both acidophilic and neutrophilic ammonia oxidizers in acidic soils by removing NO_2_^−^ before abiotic conversion to toxic compounds*.*

In this study, we investigated the cell structure, genome content, and growth characteristics of the isolate NHB1, including the effect of pH and temperature on its growth and the potential influence of NO_2_^−^ concentration in defining its pH range. As isolated acidophilic AOA are particularly sensitive to NO_2_^-^ derived compounds in culture, we tested the hypothesis that NHB1 will positively impact the growth characteristics of *Nitrosotalea* strains in coculture via continuous nitrite removal, as typically occurs in soil.

## Material and methods

### Isolation and maintenance of NHB1 in culture

*Nitrosospira* sp. AHB1 and *Nitrobacter* sp. NHB1 were previously isolated in 1988 from pH 3.8 heathland soil (Hoorneboeg; 52^o^15′N, 5^o^10′E) under *Calluna vulgaris* (Heather) and *Deschampsia flexuosa* (Wavy hair-grass) vegetation [13] and cryopreserved as a coculture which enhanced long-term stability and ease of resuscitation (data not shown). Cells were resuscitated by growing in 50 ml unbuffered medium with 2.5 mM NH_4_^+^ adjusted to pH 7 with Na_2_CO_3_ solution. Specifically, the medium contained (l^−1^): KH_2_PO_4_ (0.1 g); NaCl (0.5 g); MgSO_4_∙7H_2_O (0.04 g); CaCl_2_∙2H_2_O (0.02 g); (NH_4_)SO_4_ (0.33 g); FeSO_4_∙7H_2_O (2.46 mg); NaMoO_4_.2H_2_O (0.1 mg); MnCl_2_ (0.2 mg); Na_2_EDTA (3.31 mg); ZnSO_4_∙7H_2_O (0.1 mg), CuSO_4_∙5H_2_O (20 μg), and CoCl_2_ (2 μg) [26]. Cultures were incubated in the dark at 25°C without shaking. The same medium was subsequently used to purify NHB1, except the pH was reduced to 5.5 with HCl and (NH_4_)_2_SO_4_ replaced with 500 μM NaNO_2_ (0.035 g l^−1^). Isolation was achieved after 2 months by routine transfer (approximately every 2 weeks) of 1 ml exponentially growing culture into 50 ml fresh medium. The purity of NHB1 was initially confirmed by the absence of ammonia oxidation activity when inoculated into fresh (NH_4_)_2_SO_4_-containing medium, loss of the band corresponding to the 16S rRNA gene amplicon of *Nitrosospira* sp. AHB1 in denaturing gradient gel electrophoresis analysis, and phase contrast microscopy after four transfers. Purity was also confirmed by the absence of any contaminating DNA during genome sequencing and assembly.

The suitability of an acidophilic AOA medium for growing NHB1 in potential coculture experiments was assessed using NO_2_^−^ instead of NH_4_^+^ as an energy source. Specifically, acidic “freshwater medium” (FWM) [8] contained (l^−1^): NaCl (1 g), MgCl_2_ (0.4 g), CaCl_2_ (0.1 g), KH_2_PO_4_ (0.2 g), KCl (0.5 g), 1 ml modified nonchelated trace element solution [27], 1 ml 7.5 mM NaFeEDTA, 2 mM NaHCO_3_, and 500 μM NaNO_2_ (replacing 500 μM NH_4_Cl), was buffered with 10 mM 2-(N-morpholino)ethanesulfonic acid (MES) and adjusted to pH 5.5 with HCl before sterilization by filtration using a GL45 bottle-top 0.2 μm filter unit (Nalgene, Rochester, USA). Fifty millilitres of fresh media were inoculated with 2% (vol/vol) of the exponentially growing culture. Successful growth of NHB1 in this medium was achieved at the first attempt, resulting in its subsequent use for routine growth and maintenance.

### Ammonia, nitrite, and nitrate concentration measurements

All inorganic nitrogen concentrations were measured colourimetrically in 96-well plates using growth medium or adequate dilutions as described previously [28]. Briefly, NH_4_^+^ concentration was determined using the indophenol method [29] with 50 μl of sample. NO_2_^−^ and NO_3_^−^ concentrations were determined using a method modified from Shinn [30] and Doane and Horwath [31]. Briefly, NO_2_^−^ concentration was determined by adding 60 μl diazotizing reagent (0.3 M sulphanilamide in 3.3 M HCl) to a 100 μl sample, followed by 20 μl coupling reagent [0.12 mM N-(1-naphthyl)-ethylenediamine in 0.12 M HCl] before measuring absorbance at 540 nm. For NO_3_^−^ determination in the same sample, 20 μl vanadium chloride solution (0.4 M VCl_3_ in 1 M HCl) was then added and incubated for 90 min at 35°C in the dark to reduce NO_3_^−^ to NO_2_^−^ with the subsequently measured NO_2_^−^ concentration representing NO_2_^−^ + NO_3_^−^.

### Characterization of NHB1 growth

To determine temperature, pH, and NO_2_^−^ concentration range, triplicate 50 ml cultures were statically incubated in sterile 100 ml culture bottles in the dark between 4°C and 35°C, in medium adjusted to pH 3.5–8.0, and containing initial NO_2_^−^ concentrations between 20 μM and 10 mM, respectively. As NO_2_^−^ is known to degrade abiotically, particularly at pH lower than 5.5, triplicate sterile controls were also incubated with each treatment. All cultures were inoculated with 2% (vol/vol) early stationary phase culture that had consumed 500 μM NO_2_^−^. The growth of cultures was monitored via regular assessment of NO_2_^−^ concentrations. As NO_2_^−^ was stoichiometrically converted to NO_3_^−^ by NHB1 (Fig. S1), measurement of NO_3_^−^ concentrations was not performed on a regular basis, but NO_3_^−^ production was calculated via NO_2_^−^ consumption after calculating the predicted abiotic degradation rate of NO_2_^−^, particularly under acidic pH (Fig. S2), enabling calculation of *μ_max_* during the exponential growth phase of the cultures (Fig. S3).

### Growth of *Nitrosotalea* strains in isolation or coculture with NHB1

Cultures of isolated *N. devaniterrae* Nd1 and *N. sinensis* Nd2 or stable cocultures of *N. devaniterrae* Nd1 or *N. sinensis* Nd2 with NHB1 were established by inoculating 1 ml of early stationary phase cultures into 50 ml standard FWM medium for acidophilic AOA (pH 5.2) containing 500 μM NH_4_Cl in sterile 100 ml culture bottles. While *N. devaniterrae* Nd1 and NHB1 have similar temperature ranges and growth optima, *N. sinensis* Nd2 grows optimally ~35°C [19] and above the temperature range of NHB1. A temperature of 25°C was therefore used for isolates and coculture experiments. Growth of (co-)cultures was monitored via measurement of NH_4_^+^, NO_2_^−,^ and NO_3_^−^ concentrations until the stationary phase. The effect of pH and substrate concentration was determined in 30 ml sterile plastic Universal containers (Greiner Bio-One, Les Ulis, France) filled with 20 ml of medium. Cocultures can be maintained indefinitely by transfer (2% vol/vol) every 2–3 weeks into sterile medium. To examine the potential cyanase activity of NHB1 in coculture experiments, ammonium was substituted with 50 or 500 μM cyanate. In experiments examining yields of NO_3_^−^ with higher NH_4_^+^ concentrations, higher concentrations of MES (≥20 mM) were required to sustain growth after consumption of ~800 μM NH_4_^+^.

### Determination of substrate kinetics

Cellular nitrite oxidation kinetics were determined from instantaneous substrate-dependent oxygen uptake measurements as previously described using a multiple injection method [32, 33]. Briefly, measurements were performed in a water bath at 25°C with a microrespirometry (MR) system, equipped with a PA2000 picoammeter and a 500 μm tip diameter OX-MR oxygen microsensor (Unisense, Aarhus, Denmark), polarized continuously for at least 24 h before use. Active NHB1 cells were taken from early stationary-phase cultures soon after substrate depletion or harvested and concentrated (6000 × *g*, 10 min, 20°C) from NO_2_^−^ replete active cultures using Amicon Ultra-15 10 kDa cut-off centrifugal filter units (Merck Millipore, Darmstadt, Germany). Concentrated cells were washed with and resuspended in substrate-free medium (pH 6) prior to MR measurements. Whole cell activity rates of NHB1 were fit with the Michaelis–Menten model to derive the apparent whole cell reaction half saturation concentration (*K*m_(app)_; μM NO_2_^−^). The *K*m_(app)_ of NHB1 was compared with other nitrite oxidizer strains using previously published values [34–37].

### Transmission electron microscopy

NHB1 cells were recovered from 1 l of late exponential culture by filtering onto a 0.2 μm mixed cellulose ester filter before resuspending cells in 2 ml fresh medium and pelleting by centrifugation at 14 000 × *g* for 40 min. One volume (500 μl) 4% glutaraldehyde (vol/vol) was added to ~500 μl medium overlying the pellet and stored at 4°C overnight. Cells were then postfixed with 1% OsO_4_ in 0.3 M cacodylate buffer (pH 7.4) for 1 h at 4°C before ethanol dehydration and transfer to propylene oxide. Cells were embedded in Epon epoxy resin and inclusion obtained by polymerization at 60°C for 72 h. Ultra-thin sections (100 nm) were cut using a UC7 ultramicrotome (Leica, Nanterre, France), mounted on 200 mesh copper grids (EMS, Hatfield, USA), and contrasted with uranyl acetate and lead citrate. Sections were examined with a JEM-1400 120 kV transmission electron microscope (Jeol, Tokyo, Japan) equipped with an Orius 1000 camera (Gatan, Pleasanton, USA) in the wide-field position and Digital Micrograph software (Gatan) at the Centre d’Imagerie Quantitative Lyon-Est, Université Claude Bernard Lyon 1.

### Genome sequencing, assembly, and annotation

DNA was extracted from pelleted cells recovered from 1 l of stationary NHB1 culture using a standard sodium dodecyl sulfate buffer and phenol:chloroform:isoamyl alcohol chemical lysis method [38] and sequenced using in-house MiSeq (Illumina, San Diego, USA) and MinION (Oxford Nanopore, Oxford, UK) platforms. A library for MiSeq paired-end sequencing was prepared using a Nextera XT kit, according to the manufacturer’s instructions (Illumina) and sequencing performed with a V2 kit, producing 20.1 million reads with an average length of 243.4 bp after quality trimming performed using TrimGalore (https://github.com/FelixKrueger/TrimGalore). For MinION sequencing, DNA was sheared to ~8 kb using a g-TUBE (Covaris, Brighton, UK) and a library prepared with the SQK-MAP006 kit, according to the manufacturer’s instructions (Oxford Nanopore). Sequencing on a MinION flow cell produced 39 064 reads with an average length of 6739 bp. The genome was assembled using Unicycler [39] with default settings for both MinION and Illumina data. A unique bin was generated using MetaWRAP version 1.2.1 [40] and taxonomically annotated using GTDB-Tk version 2.1.1 with the Genome Taxonomy Database (r207) [41]. Plasmid sequences were identified using geNomad end-to-end function [42]. The GC mol% content was calculated using Seqkit fx2tab function [43]. Gene prediction was performed using Prodigal version 2.6.3 [44] and annotation performed using Diamond BLASTp v0.8.36 (*e*-value <10^−5^) [45] with the National Center for Biotechnology Information (NCBI) nr database release 244 [46]. Genome quality was assessed using CheckM [47], estimating 99.1% completeness and 0.34% contamination, and GToTree [48], detecting 100% of expected single-copy genes with 0% redundancy. To investigate the metabolic potential of NHB1, a curated protein dataset of the *Nitrobacter hamburgensis* X14 genome was downloaded from NCBI. Protein sequences were compared using Diamond BLASTp v0.8.36 (*e*-value <10^−5^) [45] to identify homologous metabolic genes. Genomic relatedness among different *Nitrobacter* strains was evaluated by calculating both Average Nucleotide Identity (ANI) and Average Amino Acid Identity (AAI) using FastANI v1.33 [49] and FastAAI [50], respectively.

### Distribution of NHB1-like *Nitrobacter* in soils

To assess the occurrence of NHB1 in soil environments, the region of the 16S rRNA gene sequence corresponding to positions 533–786 (*Escherichia coli* numbering) amplified by primers F515/R806 [51] was used as a query for read mapping against a collection of soil-derived 16S rRNA amplicon datasets (Sequence Read Archive accession numbers ERP016586, ERP016926, ERP020023, ERP020539, ERP021540, and ERP021542) previously used in a global survey of microbial diversity [52]. Mapping was performed using BBMap v38.96 [53] with a strict identity threshold of 100%.

### Phylogenomic analysis

Single-copy genes present in all compared genome sequences were identified and aligned using GToTree [48] before manual refinement. Maximum likelihood analysis was performed on unambiguously aligned concatenated protein sequences (11 751 amino acid positions inferred from 63 single-copy genes) using PhyML [54] with automatic model selection [Q.plant with FreeRate variation (four rates) across sites] and bootstrap support (100 replicates).

## Results and discussion

### Isolation and morphology of NHB1

**Figure 1:**
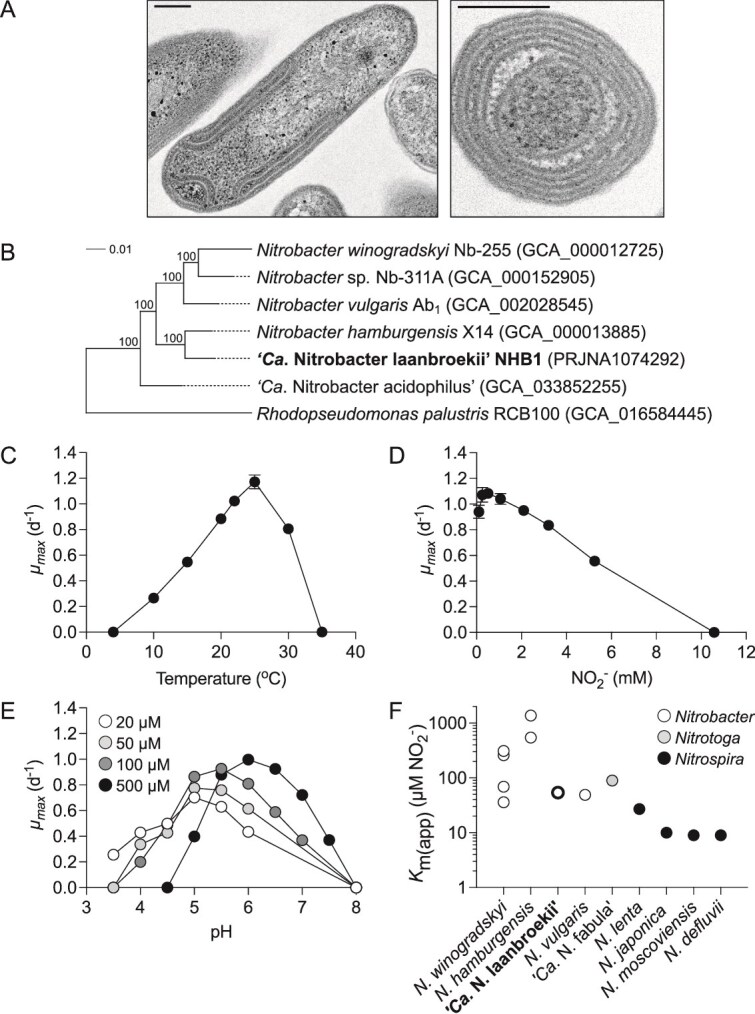
Characterization of the isolated strain “*Ca.* Nitrobacter laanbroekii” NHB1. (A) Transmission electron micrograph of cells in lateral and longitudinal orientation. Scale bars represent 0.2 μm. (B) Maximum likelihood phylogenetic tree of NHB1, selected *Nitrobacter* isolates and outgroup reference *R. palustris* (also of the *Nitrobacteraceae*) using 11 751 unambiguously aligned amino acid positions inferred from 63 single-copy genes. The scale bar represents an estimated 0.01 changes per position, and values at nodes describe percentage bootstrap support (100 replicates). The influence of temperature (at 500 μM initial nitrite concentration) (C), initial nitrite concentration (at 25°C) (D), and pH (at different initial nitrite concentrations) (E) on maximum specific growth rates (*μ_max_*) was determined with mean values and standard errors (mostly smaller than symbol size) from triplicate cultures plotted. (F) Apparent half-saturation constant [*K*_m(app)_] for NO_2_^−^ of “*Ca*. Nitrobacter laanbroekii” NHB1 (mean value of four replicates) and various *Nitrobacter, Nitrotoga,* and *Nitrospira* strains.

NHB1 was isolated from a cryopreserved coculture of NHB1 and AHB1 by substituting NH₄Cl in the growth medium with NaNO₂. As with all *Nitrobacter* strains, NHB1 exhibits a characteristic rod-shaped morphology [55]. It features the typical stacked intracytoplasmic membranes, arranged as a polar cap of paired membranes. These membranes contain nitrite oxidoreductase, an essential enzyme for nitrite oxidation in *Nitrobacter* strains (Fig. 1A) [56].

### Genome analysis of NHB1

NHB1 possesses a 3.3 Mb chromosome and two plasmids (0.28 and 0.21 Mb), with an overall GC content of 62.3%. Its chromosome is notably smaller than those of other *Nitrobacter* strains isolated from diverse environments [20, 22, 57] (Table 1), particularly in comparison to its closest validated relative, the neutrophilic *N. hamburgensis* X14 [58] (Fig. 1B). Despite this 24% reduction in genome size relative to *N. hamburgensis* X14, NHB1 shares 99%, 97%, and 98% identity in the 16S rRNA, *nxrA*, and *nxrB* genes, respectively, with high identity between marker genes common in comparisons with *Nitrobacter* species [58]. Whole-genome comparison reveals that NHB1 shares an ANI and AAI of 92.4% and 86.5% with *N. hamburgensis* X14, respectively, and lower with other validated *Nitrobacter* species (Table 1).

**Table 1:** Comparative genome characteristics of “*Ca.* N. laanbroekii” NHB1 with selected validated *Nitrobacter* species.

Strain	Origin	Chromosome size (Mb)	GC (%)	ANI (%)	AAI (%)
*Ca*. Nitrobacter laanbroekii NHB1	Soil	3.3	62.3	-	-
*Nitrobacter hamburgensis* X14	Soil	4.4	61.6	92.4	86.5
*Nitrobacter winogradskyi* Nb-255	Soil	3.4	62.0	84.7	74.5
*Nitrobacter* sp. Nb-311A	Marine water	4.1	62.0	84.5	75.0
*Nitrobacter vulgaris* Ab_1_	Sewage	3.9	59.8	85.1	76.8
*Ca*. Nitrobacter acidophilus	Freshwater sediment	3.9	62.3	87.0	76.4

### Metabolism of NHB1

In addition to core genes for carbon and energy/nitrite metabolism, NHB1 possesses genes for dissimilatory sulphur oxidation, assimilatory nitrite reductase, and carbon monoxide utilization, typical of those found in other *Nitrobacter* species (Table S1) [58]. For nitrite metabolism and energy conservation, NHB1 encodes the essential genes *nxrA*, *nxrB*, and *nxrC*, responsible for producing the three subunits of nitrite oxidoreductase (NXR) that belong to type II DMSO reductase-like family of molybdopterin-binding enzymes [59–61]. NHB1 has multiple copies of *nxrA* (three copies) and *nxrB* (two copies) as found with *N. hamburgensis* and *N. winogradskyi* [57, 58]. NHB1 also encodes other genes of accessory proteins related to nitrate reductase (*nxrX, nxrD*, cytochrome c class I) and typically found in the genomes of other *Nitrobacter* strains [58]. NXR is located in the cytoplasm of *Nitrobacter*, and NHB1 possesses nitrite/nitrate transporters (*narK* transporter superfamily, TDT family of transporters) to facilitate the transfer of NO₂^−^ and NO₃^−^ across the cytoplasmic membrane [4, 58]. Additionally, NHB1 exhibits assimilatory nitrogen metabolism enabling the conversion of nitrite to ammonia via assimilatory nitrite reductase (*nirBD*) [57, 58]. For urea metabolism, NHB1 lacks urea catabolic genes, similar to *N. winogradskyi* [57]. In contrast, *N. hamburgensis* encodes genes for urea carboxylase and urea/short-chain ABC transporters, which require energy for function [58].

All *Nitrobacter* species assimilate sulphur by converting sulphate to sulphide, a form that can be incorporated into the amino acid cysteine. NHB1 genome contains a group of genes associated with the dissimilatory sulphur oxidation (*soxXYZAB*) (Table S1), which closely resemble those found in *N. hamburgensis* [58].

NHB1 encodes RuBisCO, a key enzyme complex for carbon dioxide fixation via the Calvin cycle, which is conserved across all *Nitrobacter* strains [57, 58, 62]. It also encodes PII-like regulatory proteins, which are known to control nitrogen assimilation and may play a role in regulating carbon fixation or coordinating carbon and nitrogen metabolism [58]. Additionally, NHB1 contains four gene clusters encoding molybdopterin-containing carbon monoxide dehydrogenase (Mo-CODH), consistent with other *Nitrobacter* strains, although the number of clusters varies between species [58]. NHB1 carries the same number of Mo-CODH homologues as *N. hamburgensis*, including the *coxL/cutL* gene, which encodes the large subunit of the enzyme. In contrast, *N. winogradskyi* possesses only one gene cluster, which, despite its high similarity to the *N. hamburgensis* cluster, lacks the gene encoding the large subunit [57, 58]. NHB1 also encodes formate dehydrogenase (FDH), the TorD chaperone, and the enzymes required for molybdopterin cofactor biosynthesis, all of which share high sequence similarity to those found in *N. hamburgensis* [58].

### Physiological characterization of NHB1

Growth of NHB1 occurred with initial NO_2_^−^ concentrations up to 5 mM (inhibited at ~10 mM) and temperatures ranging 10°C–30°C, with a maximum specific growth rate (*μ_max_*) of 1.17 d^−1^ (s.e. = 0.05) at 25°C and 0.5 mM NO_2_^−^ (Fig. 1C and D). The optimal growth temperature of NHB1 is consistent with most *Nitrobacter* isolates (25°C–28°C) [63, 64]. As indicated from its previous cultivation, NHB1 is acidotolerant but *μ_max_* at different pH varied with initial NO_2_^−^ concentration. At 500 μM, growth occurred at pH 5.0–7.5 with optimal growth at pH 6.0 (Fig. 1E), but reduction of initial NO_2_^−^ concentration to 100 μM decreased optimal growth pH to 5.5 [*μ_max_* 0.93 d^−1^ (s.e. = 0.01)] with further reductions in pH growth optimum to 5.0 at 50 and 20 μM [*μ_max_* 0.77 d^−1^ (s.e. = 0.03) and 0.70 d^−1^ (s.e. = 0.01), respectively]. At the lowest NO_2_^−^ concentration, the limit for growth was extended to pH 3.5 [*μ_max_* 0.26 d^−1^ (s.e. = 0.01)]. In contrast, neutrophilic *Nitrobacter* strains tolerate higher NO₂^−^ concentrations, consuming ≥10 mM NO₂^−^ at an optimal pH of ≥6 [64].

Whole cell nitrite oxidation kinetics at 25°C and pH 6 were determined using substrate concentration-dependent oxygen microrespirometry (Fig. S4). An apparent-half-saturation concentration [*K*_m(app)_] of 54 μM NO_2_^−^ (s.e. = 7) was higher than several canonical nitrite-oxidizing *Nitrospira* but within the lower range of those observed for other *Nitrobacter* strains, and one order of magnitude lower than *N. hamburgensis* X14 (544 μM) [34, 35, 65] (Fig. 1F).

### Influence of NHB1 on acidophilic ammonia-oxidizing archaea physiology

**Figure 2:**
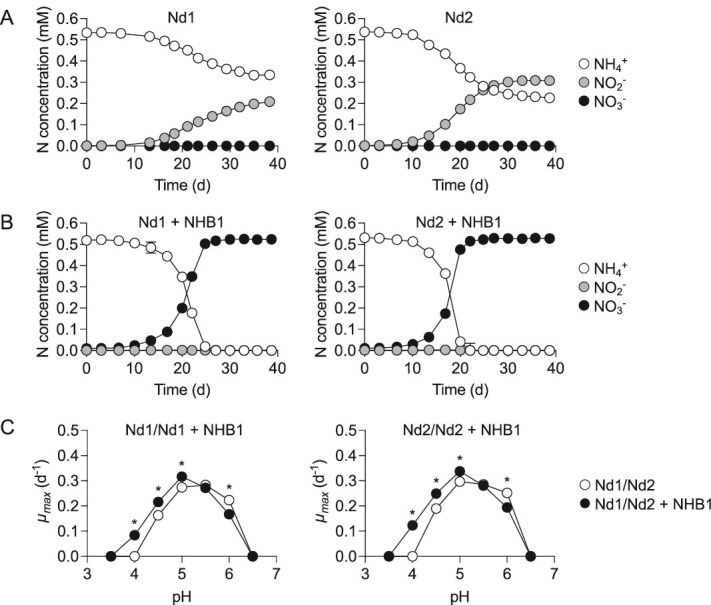
Influence of “*Ca.* Nitrobacter laanbroekii” NHB1 on growth of acidophilic AOA *N. devaniterrae* Nd1 and *N. sinensis* Nd2 in batch culture. Mean values and standard errors (mostly smaller than symbol size) from triplicate cultures are plotted in each panel. (A) Growth of Nd1 and Nd2 grown in isolation and supplied with 0.5 mM NH_4_^+^. (B) Growth of Nd1 and Nd2 in coculture with NHB1 supplied with 0.5 mM NH_4_^+^. (C) Influence of NHB1 on the maximum specific growth rates (*μ_max_*) of Nd1 and Nd2 compared to growth in isolation at different pH. An asterisk highlights a significant difference in *μ_max_* (*P* = ≤.05) for an individual pH (calculated using Student’s *t*-test).

**Figure 3:**
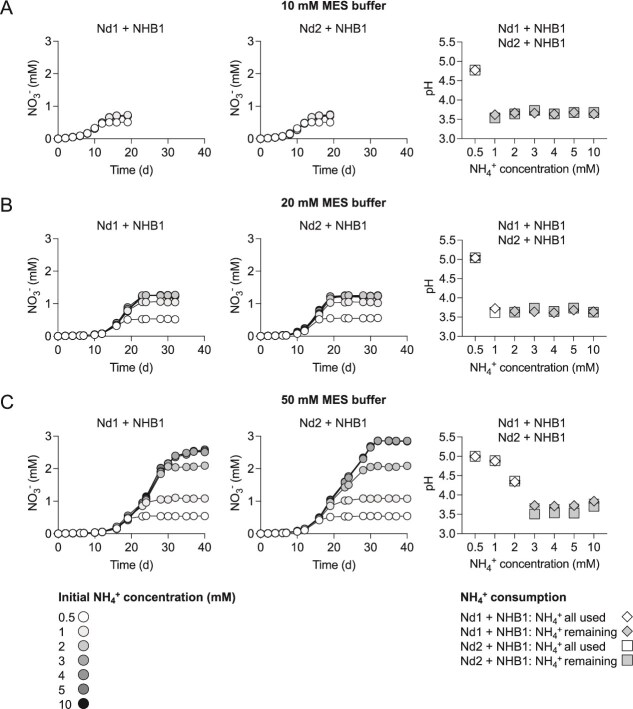
Effect of MES buffer concentration [10 mM (A), 20 mM (B), 50 mM (C)] on the growth of cocultures of “*Ca*. Nitrobacter laanbroekii” NHB1 and *N. devaniterrae* Nd1 or *N. sinensis* Nd2 in medium with an initial pH 5.2 at different NH_4_^+^ concentrations (0.5, 1, 2, 3, 4, 5, and 10 mM). NO_3_^−^ concentrations were determined through to stationary phase and pH measured at the last time point. The presence of remaining NH_4_^+^ at the end of the stationary phase is described and demonstrates inhibition by decreasing pH rather than substrate limitation. Mean values and standard errors (mostly smaller than symbol size) from triplicate cultures are plotted in all panels.

*N. devaniterrae* Nd1 and *N. sinensis* Nd2 are two AOA strains with pH optima ~5.0 [8, 9, 19]. Although obligately acidophilic with adaptations to low pH, both are sensitive to NO_2_^−^ with concentrations as low as 10 μM reducing growth rates [8, 9, 19]. In standard batch cultures at pH 5.2, yields of NO_2_^−^ are typically 200–300 μM (Fig. 2A), which is one to two orders of magnitude lower than neutrophilic soil AOA [66, 67]. However, in contrast to growth in isolation, all 500 μM NH_4_^+^ was oxidized when grown in coculture with NHB1 (Fig. 2B), avoiding NO_2_^−^ accumulation and the formation of nitrous acid (HNO₂) and its decomposition products [7]. The amount of NH_4_^+^ in the medium that could be oxidized was extended to 1.2 and 2.7–2.9 mM for both strains when MES buffer concentrations were increased from 10 to 20 and 50 mM, respectively (Fig. 3), with growth inhibited when pH eventually decreased to <3.8 (Fig. 3). In the presence of NHB1, the pH range of both AOA was extended down by ~0.5 pH units and *μ_max_* increased significantly at pH ≤5.0 (*P* = <.05) (Fig. 2C).

### Cyanase facilitates cross-feeding between NHB1 and acidophilic ammonia-oxidizing archaea

*Nitrobacter* isolates including NHB1 possess genes encoding enzymes that were previously demonstrated to facilitate mutualistic interactions with ammonia oxidizers, such as cyanase, which produces NH_4_^+^ from cyanate and enables reciprocal cross-feeding [4, 68]. Cyanate can be produced through carbamoyl phosphate metabolism and urea degradation or imported from the environment via specific transporters for the uptake of nitrite, which are encoded in the NHB1 genome [68].

**Figure 4:**
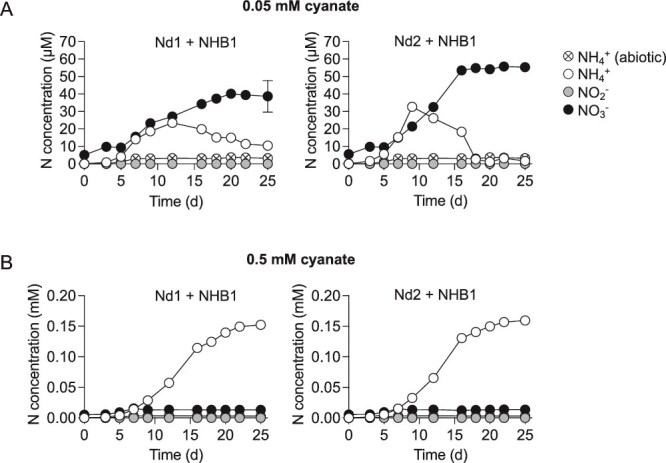
Cyanate facilitating reciprocal cross-feeding at pH 6.0 between “*Ca*. Nitrobacter laanbroekii” NHB1 and *N. devaniterrae* Nd1 or *N. sinensis* Nd2. Cyanate was supplied at 0.05 mM (A) or 0.5 mΜ (B) as an NH_4_^+^ source. NH_4_^+^, NO_2_^−,^ and NO_3_^−^ concentrations were determined for all cultures and mean values and standard errors (mostly smaller than symbol size) from triplicate cultures are plotted. Decomposition of cyanate in the abiotic control produced <3.8 μM NH_4_^+^ during the period of incubation.

The stability of cyanate is pH-dependent, with abiotic degradation to ammonium occurring more readily in acidic conditions [69]. While cyanate abiotically degraded rapidly to NH_4_^+^ in pH 5.2 medium (Fig. S5), a phenomenon that also happens in nature, NHB1 enzymatic cyanase activity was the dominant mechanism generating ammonium at pH 6.0 in cocultures supplemented with 0.05 or 0.5 mM cyanate (Fig. 4). At 0.05 mM, a concentration higher than that typically found in soil where cyanate is continuously turned over [69], rates of NHB1 cyanase activity were faster than the ammonia oxidation activity of Nd1 or Nd2, leading to NH_4_^+^ accumulation over the first 10–12 days before decreasing as the majority or all of the cyanate-derived NH_4_^+^ was oxidized to NO_2_^−^ by the AOA species and subsequently to NO_3_^−^ by NHB1. Interestingly, NO_2_^−^ concentrations were below the detection limit throughout the 25-day experiment, highlighting the differences in substrate turnover/conversion rates between cyanate, ammonia, and nitrite. Although ≥0.5 mM cyanate can support other AOA or nitrifying cocultures [68], this concentration inhibited both *Nitrosotalea* strains. At the higher concentration tested, NHB1 cyanase activity occurred without energy from NO_2_^−^ oxidation but could not be sustained. Approximately 0.15 mM of the total 0.5 mM cyanate was converted to NH_4_^+^, but <5% of NOB-generated NH_4_^+^ was oxidized through to NO_3_^−^ and with no accumulation of NO_2_^−^ (Fig. 4B).

### Presence of NHB1 in soils

To determine whether NHB1 (or NHB1-like) strains are commonly found in soil, 16S rRNA amplicon sequence collections previously used in a global meta-analysis of prokaryote diversity in various habitats were examined [52]. Using soil-only datasets (Table S2), the presence of NHB1-like strains was demonstrated by identifying the V4 region amplified sequence variant (ASV) at 100% identity and that is distinct from the equivalent ASV of *N. hamburgensis* X14 with two mismatches over the same region. Of the 145 sequenced soil samples included, 36% contained the NHB1 ASV. This included all soils sampled over two consecutive years from a contiguous, long-term pH gradient (pH 4.5, 5.0, 5.5, 6.0, 6.5, 7.0, and 7.5) of arable soil [70] with a relative abundance ranging from 0.002% to 0.02% and an average of 0.01%. These data demonstrate that NHB1-like *Nitrobacter* strains are widely distributed in soils globally and are not restricted to narrow pH ranges, consistent with the characterized pH range of NHB1 in culture observed in this study (i.e. pH 3.5–7.5).

### Summary

NHB1 is an acidotolerant NOB isolated from acidic soil with a relatively high affinity for NO_2_^−^. Growth in acidophilic consortia demonstrates that continued removal of NO_2_^−^ in acidic soil is likely crucial for sustained growth of ammonia oxidizers and that consideration of substrate and metabolic product concentrations is essential when characterizing physiology. As the ANI between NHB1 and its closest validated relative *N. hamburgenisis* X14 is less than the species cut-off of 95% ANI [49], in addition to its contrasting ecophysiology, we propose the following candidate species: *Nitrobacter laanbroekii* sp. nov. (laan.broek'i.i. N.L. gen. n. *laanbroekii*), named in honour of the Dutch microbiologist Hendrikus J. Laanbroek who was involved in its original cultivation and has made valuable contributions to understanding the ecology of NOB.

## Supplementary Material

Bachtsevani_Hink_et_al_Supplementary_Figures_ycaf244

Bachtsevani_Hink_et_al_Supplementary_Tables_ycaf244

## Data Availability

The genome sequence of “*Ca*. Nitrobacter laanbroekii” NHB1 is available under NCBI BioProject accession number PRJNA1074292.
