# Supplementary material for: Acidotolerant soil nitrite oxidizer “Candidatus Nitrobacter laanbroekii” NHB1 alleviates constraints on growth of acidophilic soil ammonia oxidizers
Source: ISME Commun. 2025 Dec 23;6(1):ycaf244. doi: 10.1093/ismeco/ycaf244 (PMC12815253; doi:10.1093/ismeco/ycaf244)
Supplement: Bachtsevani_Hink_et_al_Supplementary_Figures_ycaf244 [file bachtsevani_hink_et_al_supplementary_figures_ycaf244.pdf]

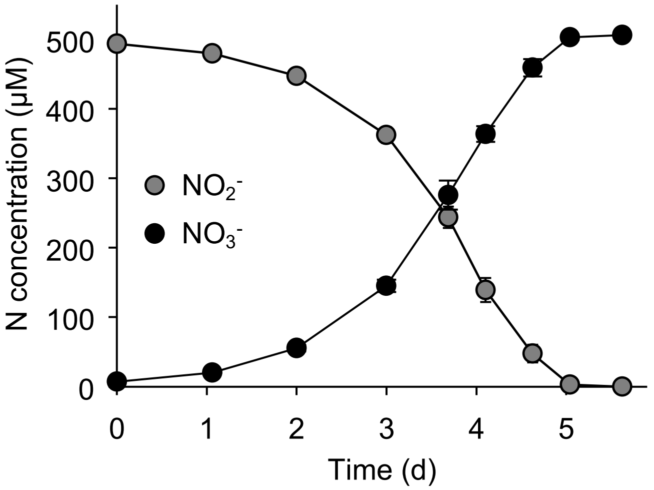

Figure S1. Stoichiometric relationship of NO_2^- consumption and NO_3^- production during growth of '*Ca.* Nitrobacter laanbroekii' NHB1. The medium was adjusted to pH 5.5 with an initial NO_2^- concentration of 500 µM and inoculated with 1% early stationary culture (vol/vol) and incubated at 25°C in the dark. Plotted values are the mean and standard errors of NO_2^- and NO_3^- concentrations of triplicate cultures. The confirmed stoichiometry between NO_2^- consumption and NO_3^- production enabled the use of NO_2^- concentrations to infer NO_3^- concentrations in subsequent culture characterisation experiments.

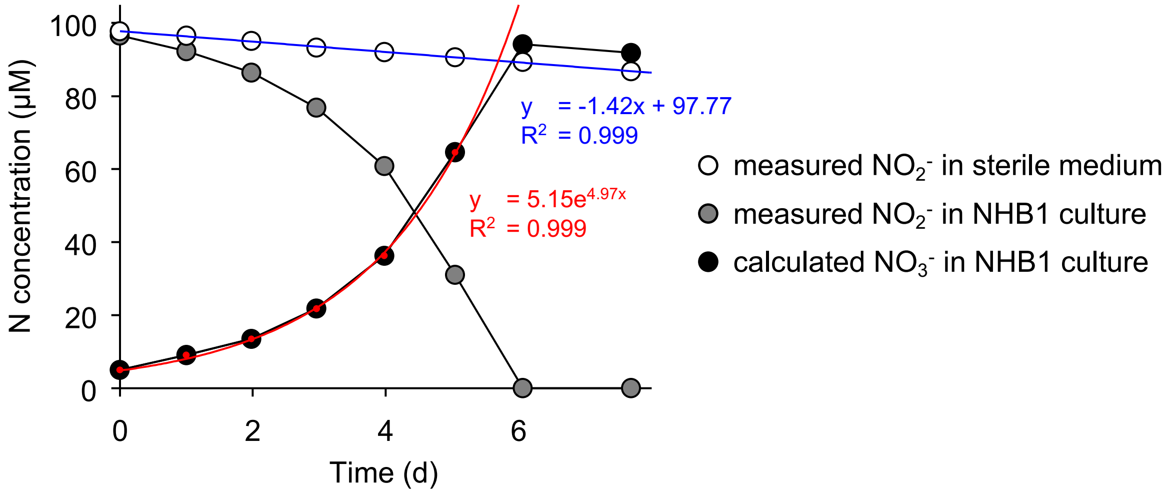

Figure S2. An example of assessing μ_{max} of '*Ca.* Nitrobacter laanbroekii' NHB1 when grown at pH 4.5 with an initial 100 μM NO_2^- concentration. Medium was inoculated with a 1% transfer (vol/vol) of an early stationary culture that had consumed 500 μM NO_2^-. Sterile medium served as control to monitor abiotic NO_2^- degradation following a linear decline over time (blue regression and equation). Measured NO_2^- concentrations in the growing culture and the abiotic degradation rate NO_2^- in the sterile control was used to calculate predicted NO_3^- concentrations. An exponential curve was then fitted to the NO_3^- data during exponential growth of the culture (red circles, regression and equation). The parameter in the exponent of the equation corresponds to the μ_{max} value of the culture.

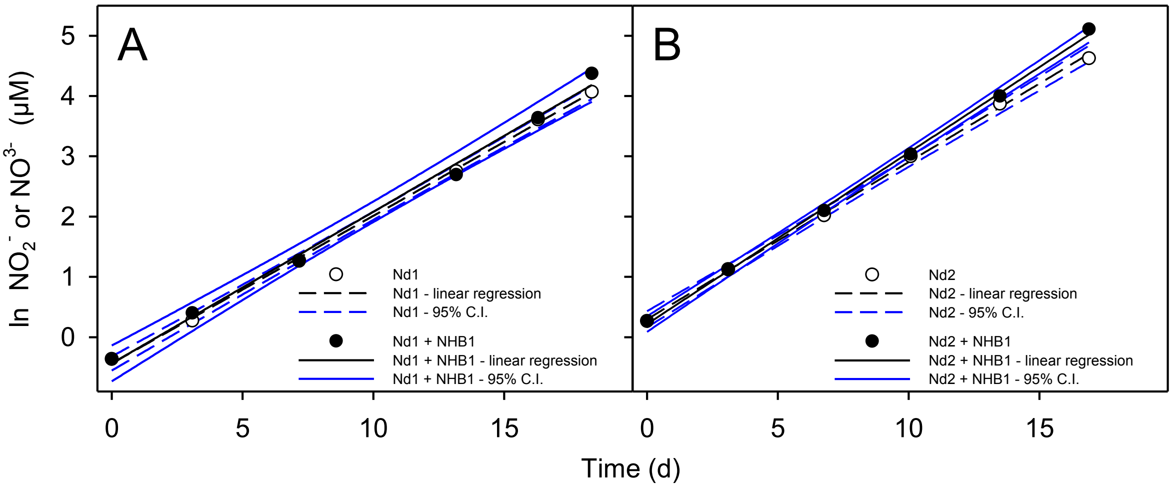

Figure S3. Examples of plots used for calculating maximum specific growth rates of (A) *Nitrosotalea devaniterrae* Nd1 and (B) *Nitrosotalea sinensis* Nd2 (assessed via NO_2^- production) or in co-culture with '*Ca.* Nitrobacter laanbroekii' NHB1 (assessed via NO_3^- production) in medium supplied with 500 µM NH_4^+. Linear regression and 95% confidence intervals (C.I.) were calculated from natural logarithm (ln) values of NO_2^- concentrations during the exponential phase of growth.

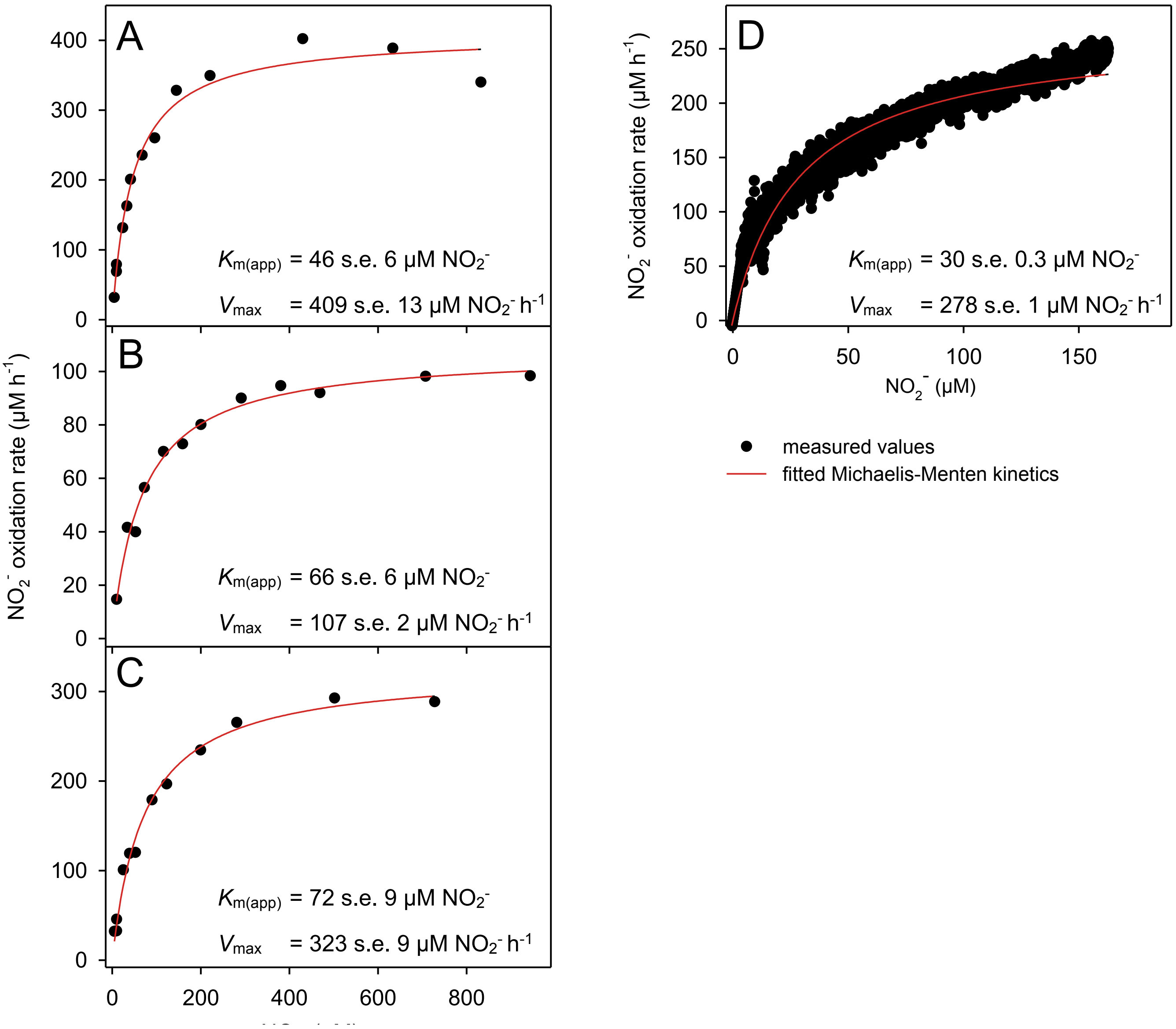

Figure S4. Apparent half-saturation ($K_{m(app)}$) and maximum oxidation rates (V_{max}) for NO_2^- calculated using oxygen microrespirometry (MR) after fitting data of oxidation rate vs NO_2^- concentration to the Michaelis–Menten model plotted. Three experimental replicates measured oxidation rates after injection varying concentrations of NO_2^- (A,B,C) into the MR chamber and one replicate (D) used a single injection of 250 μm NO_2^-. Biomass was not measured and V_{max} was therefore not normalised to cell numbers or protein content and varies substantially amongst replicates.

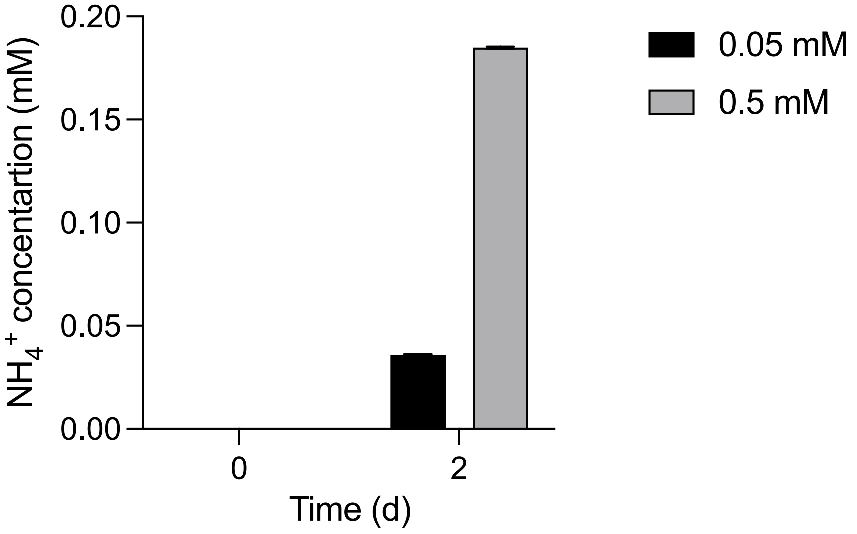

Figure S5. Production of NH_4^+ from abiotic decomposition of cyanate in pH 5.2 'freshwater medium' amended with 0.05 mM or 0.5 mM after two days incubation at 25°C.
